# Abundance of badgers (*Meles meles*) in England and Wales

**DOI:** 10.1038/s41598-017-00378-3

**Published:** 2017-03-21

**Authors:** Johanna Judge, Gavin J. Wilson, Roy Macarthur, Robbie A. McDonald, Richard J. Delahay

**Affiliations:** 1 National Wildlife Management Centre, Animal and Plant Health Agency, Woodchester Park, Gloucestershire, GL10 3UJ UK; 2 National Biodiversity Network, Broadway Business Centre, 32a Stoney Street, Lace Market, Nottingham, NG1 1LL UK; 3 Biocensus Limited, The Malt House, 17–20 Sydney Buildings, Bath, BA2 6BZ UK; 4 FERA Science Ltd., Sand Hutton, York, YO41 1LZ UK; 5 Environment and Sustainability Institute, University of Exeter, Penryn Campus, Penryn, TR10 9EZ Cornwall, UK

## Abstract

The European badger (*Meles meles*) is of considerable interest in the UK as it is both a protected species and the main wildlife reservoir for bovine tuberculosis infection in cattle. While there have been three national badger surveys in the 1980s, 1990s and 2011–13, using the number of badger main setts as a proxy for the abundance of badger social groups, none has combined contemporary data on social group size at landscape and national scales. We estimated social group size by genotyping hair samples collected at 120 main setts across England and Wales and employing a capture-mark-recapture method based on genotypes. The estimated mean social group size in England and Wales was 6.74 (±0.63) badgers. There was considerable variation in badger social group size among Land Class Groups (LCGs), with a low of 2.67 in LCG3 and a high of 7.92 in LCG4. Combining these results with the recent Badger Sett Survey of England and Wales, we estimate there are approximately 485,000 badgers (95% confidence intervals 391,000–581,000) in England and Wales. Although direct comparison with previous estimates is not ideal owing to methodological differences, our results are consistent with a marked increase in the badger population of England and Wales since the 1980s.

## Introduction

The European badger (*Meles meles*) has been the focus of significant research interest and political debate in the United Kingdom. On the one hand, the badger is an icon of conservation and, owing to a history of persecution, is protected by U.K. and international legislation. On the other hand, badgers contribute to the persistence of bovine tuberculosis in the U.K. cattle population^1^ and are involved in damage to crops, buildings and infrastructure^2, 3^, thus raising licensing and management issues for U.K. governments. The challenge for researchers is how to generate reliable estimates of badger abundance at both regional and national scales, to inform decisions on potential management options.

Direct estimation of badger numbers is challenging owing to their nocturnal and fossorial habits. However, as badgers live in territorial social groups across much of their UK range and occupy relatively conspicuous burrows (setts), this provides opportunities for indirect estimation of abundance. As a general rule each badger social group would be expected to have one “main” sett in their territory, which is occupied throughout the year and forms the focus for most social interaction and breeding. They also have other, generally smaller and more intermittently used, subsidiary and outlying setts elsewhere in the territory^4^. Hence main setts provide opportunities for estimating numbers of badger social groups. Two large scale surveys of badger setts in England, Wales and Scotland^5, 6^ have used the presence of main setts as a proxy for the presence of badger social groups to provide estimates of badger social group abundance in Great Britain. More recently, further sett surveys have been undertaken in Scotland^7^, Northern Ireland^8^ and England and Wales^9^ all of which provided updated estimates of badger social group abundance and have outlined significant changes in the abundance of badger social groups.

Social group abundance does not directly equate to badger abundance, but it could be used to estimate badger abundance, if reliable data on social group sizes were available. Previously, estimates of badger population size have used published data from a small range of studies as a multiplier to derive population estimates from sett survey data^5, 6^. However, these studies were not all contemporary with, nor fully representative of the geographical area or landscapes covered by, the sett surveys. These are potentially important shortcomings as badger social group size is known to vary considerably both in space and time^10–12^. The Woodchester Park long-term study of a high-density badger population has identified substantial variation in the number of animals present within and among social groups^13^. Furthermore, in this same population, changes in badger abundance were driven by variation in social group size whilst the total number of groups and associated main setts remained relatively stable^14^. By contrast, in a long-term study of badgers in Sussex, the number of main setts and associated badger social groups more than doubled over a 20 year period, while social group territory sizes halved^15^. Another long-term study, at Wytham Woods in Oxfordshire, described how the number of badger social groups in the study area increased from 1974 to 1993, but that subsequent growth of the population took place in the absence of further changes in the number of social groups^16^. Hence, a change in the density of main setts may not necessarily be accompanied by a proportional change in badger abundance. Furthermore, at a larger geographic scale there is some evidence that social group size is typically smaller in lower quality habitats^17^ suggesting that in order to estimate badger abundance at such scales, social group size may need to be estimated across a range of landscape types.

In the present study, we conducted hair trapping and genotyping to generate contemporary estimates of mean badger social group sizes for areas of England and Wales that had been part of our 2011–13 badger sett survey^9^. We estimated badger social group sizes using capture-mark-recapture analyses of the genotyped hair. We then combined our estimates of social group size and abundance to produce robust estimates of the abundance of badgers in England and Wales.

## Results

A total of 635 hair traps were placed at 122 main setts (average 8.2 traps/sett; range 2 to 18) in 87 × 1 km squares (range 1–3 setts/square) distributed among Land Class Groups 1–6. During the 2011–13 badger sett survey of England and Wales^9^ no main setts were recorded in any of the 29 squares surveyed in Land Class Group 7, therefore, it was not possible to set hair traps in this Group. Between one and 126 (mean = 27.6) hair samples were collected per sett. In total 3362 hair trap-day samples were collected and submitted for analysis. Full genetic profiles were returned from 1415 (41.9%) of these samples and a total of 501 individuals were identified. The number of individual badgers per social group, estimated by capture-mark-recapture analysis of genotypes varied within and between Land Class Groups (LCG) (Table 1, Supplementary Figure S1). The mean estimated number of badgers per social group varied from 2.67 in LCG3 to 7.92 in LCG4 (Table 1).

**Table 1:** Estimates of the mean number of badgers per social group in each Land Class Group, the total number of social groups sampled in each Land Class Group and the number of those social groups at which only one badger was identified.

Land Class Group	Number of social groups	Number of single capture social groups	Estimated mean number of badgers/social group	Standard error of estimate
1	13	1	6.54	1.38
2	15	0	6.27	1.74
3	3	0	2.67	0.27
4	38	2	7.92	0.86
5	8	0	7.50	1.72
6	12	1	4.17	0.57

Land Class Group 7 was not included because no main setts were recorded in any of the surveyed squares in this Group during the 2011–13 sett survey.

**Figure 1:**
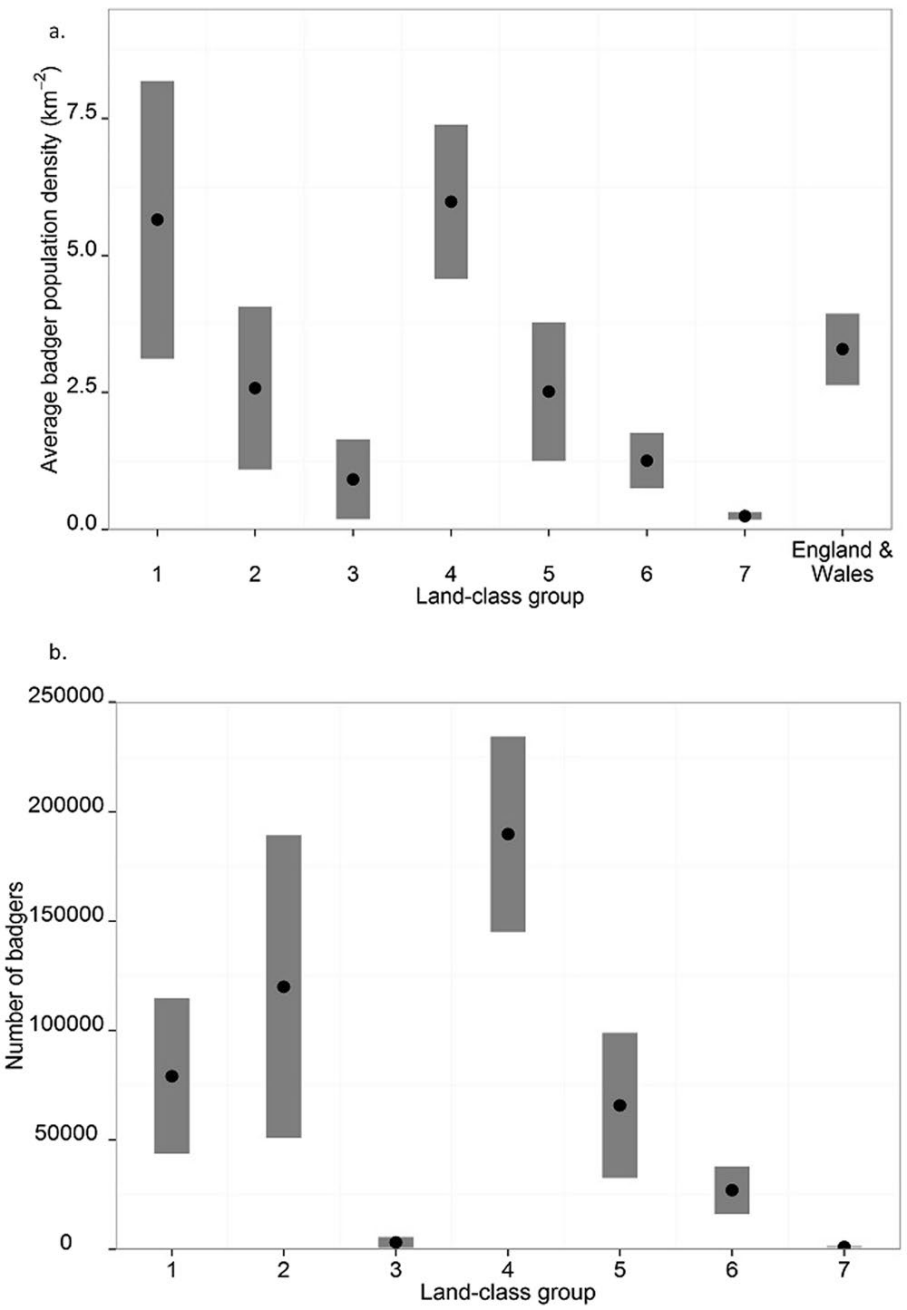
Estimates of badger population size (**a**) and density (**b**) in each Land Class Group. Shaded blocks indicate 95% confidence intervals for means.

Combining the estimated mean number of badgers per social group in each LCG derived from surveys in 2012–14 with the mean number of badger social groups per LCG derived from the badger sett survey conducted in 2011–13^9^ results in a total population estimate of approximately 485,000 badgers (95% confidence intervals 391,000–581,000) in England and Wales (Table 2, Fig. 1). Of these 55% are in LCGs 1 and 4, which are lowland pastoral landscapes predominantly found in the south-west of England and south Wales.

**Table 2:** The estimated number of badgers in each Land Class Group as calculated from the estimated mean number of badgers per social group and number of social groups and extrapolated population estimates for England and Wales combined (with standard errors, relative standard errors and 95% confidence intervals shown as appropriate).

Land Class Group	Mean badgers per social group			Number of Groups			Number of badgers		
	estimate	se	rse (%)	estimate	se	rse (%)	estimate	se	95% CI
1	6.54	1.38	21.1	12110	979	8.1	79179	17890	±35065
2	6.27	1.74	27.8	19155	1557	8.1	120037	34714	±68040
3	2.67	0.27	10.2	1188	466	39.2	3169	1284	±2517
4	7.92	0.86	10.8	23951	1250	5.2	189716	22779	±44647
5	7.50	1.72	23.0	8769	1071	12.2	65769	17100	±33517
6	4.17	0.57	13.7	6472	983	15.2	26965	5508	±10795
7	4.17^a^	0.57^a^	13.7^a^	274^b^	NA	NA	1140	156	±305
England & Wales	6.74	0.63	9.4	71919	2697	3.8	484714	48653	±95360

^a^Assumed to be equal to LCG 6, ^b^based on the observed number of other setts and the assumption that there is approximately one main sett for every four “other” setts in LCG7.

Using the total number of 1 km squares in each LCG and in the whole of England and Wales, badger population density in England and Wales combined was estimated to be 3.29/km^2^. Mean badger density estimates per LCG ranged from 0.26/km^2^ (LCG7) to 5.98/km^2^ (LCG4) (Table 3, Fig. 1). The expected badger population size is approximately 424,000 in England and 61,000 in Wales.

**Table 3:** Estimates of mean badger population density in each Land Class Group with standard error and 95% confidence intervals.

Land Class Group	Total number of 1-km squares	Mean population density		
		estimate	se	95% CI
1	14006	5.65	1.28	±2.50
2	46558	2.58	0.75	±1.46
3	3461	0.92	0.37	±0.73
4	31723	5.98	0.72	±1.41
5	26159	2.51	0.65	±1.28
6	21453	1.26	0.26	±0.50
7	4380	0.26	0.04	±0.07
England & Wales	147738	3.29	0.33	±0.65

## Discussion

This study represents the first systematic attempt to generate contemporary badger social group size estimates across the range of landscapes found in England and Wales. Our results show substantial variation in group size within and among Land Class Groups^10–12^. Combining our estimates of badger social group sizes with the estimated number of social groups from the recent sett survey of England and Wales^9^ yielded an estimate of the total badger population in England and Wales in 2011–14 of 485,000 (95% confidence interval 391,000–581,000), and an overall density of 3.29 badgers/km^2^ (95% CI 2.64–3.94).

The uncertainty associated with the population size estimate presented here is approximately ±20% and has three main sources. First, there is variation in the number of social groups observed in each location surveyed within each LCG^9^. Second, there is variation in the number of individuals present in each social group within each LCG. The third source of uncertainty is associated with the estimated number of individuals within each social group. Each of these sources of variation contributes to uncertainty about the average badger population density within each LCG. Because of the way in which the effect of independent sources of variation or uncertainty combine, smaller sources of uncertainty make a disproportionately small contribution to the total uncertainty. This means that just looking at the size of the individual sources of uncertainty can give a misleading impression about their relative importance. Hence, we estimated how much the uncertainty associated with the national estimate could be reduced if these sources of variation were reduced to zero. For example, surveying a much larger area of land would reduce uncertainty associated with the average density of social groups. We estimated that this could result in a modest reduction in uncertainty associated with the total population size estimate from ±19.7% to ±18.3%. Similarly surveying each group much more intensively is expected to reduce the uncertainty associated with the estimated total population size from ±19.7% to ±19.3 whereas greatly increasing the number of social groups surveyed is estimated to reduce the uncertainty associated with the estimated total population size from ±19.7% to ±8.2% Therefore, all other things being equal, the greatest reduction in uncertainty in the population estimate would be achieved by surveying a larger number of social groups to improve the estimated average number of badgers per group. Furthermore, it would not be prudent to increase the area of land surveyed for badger setts or the intensity with which groups were surveyed without also increasing the number of social groups surveyed because the total uncertainty would not be reduced.

In future, the precision of national estimates of badger abundance could best be improved by deploying more effort to collect social group size data from a larger sample of groups. Therefore in any repeat survey, there may be a requirement to consider relative deployment of effort between sett surveys and social group size estimation. Exactly how this is done will depend on the aims of a follow-up survey, and the available resources. For example, if the aim is to detect change in abundance of the national badger population, then optimising precision by increasing the sample of social group size estimates may be necessary. Alternatively, understanding changes in the distribution of badgers may best be achieved by focussing resources on sett surveys.

One previous study attempted to quantify the size of the national badger population by using an overall estimate of social group size of 5.9, derived from live-capture results from field studies based largely in south-west England^5^. This resulted in an estimated badger population in Great Britain (i.e. England, Wales and Scotland) in the 1980s of approximately 250,000. The wide variation in social group sizes observed in the present study suggests that the use of a single group size multiplier derived largely from one region is almost certain to result in biased population estimates. While it is not possible directly to compare that population estimate with our findings owing to this difference in social group size methodology the results of the most recent badger sett surveys of England and Wales^5, 9^ which used directly comparable approaches, suggested an 88% increase in the abundance of badger social groups in the two countries between the 1980s and 2011–14. As the badger population estimate presented here is almost double the previous estimate for the whole of Great Britain^5^, and assuming that social group sizes were not grossly overestimated in the present study, the evidence suggests that the badger population in England and Wales has increased substantially since the 1980s. Importantly, the results of the present study provide a robust contemporary estimate of the size of the badger population in England and Wales derived from a representative and systematic sampling approach. This provides a population baseline against which subsequent changes in badger abundance in England and Wales could be directly compared.

## Methods

### Sampling Design

The Land Classification System was devised by the Centre for Ecology and Hydrology (CEH) and it assigned each 1 km square in the UK to one of 32 Land Classes according to its predominant habitat type^18, 19^. The CEH system was simplified into seven broad landscape types, known as Land Class Groups (LCG1 to LCG7; see Judge *et al.* ^9^ for further details), as used in previous stratified surveys of mammals^20–22^ including badgers^5, 6^ and in the 2011–13 badger sett survey^9^.

The two previous badger sett surveys of England and Wales^5, 6^ demonstrated that variation in the number of main setts (and, therefore, social groups) per square was greater in some LCGs than others. Therefore, the most recent sett survey^9^ adopted a disproportionate stratified random sampling strategy, in order to focus on those LCGs with the highest expected variation, which were also the squares with historically higher badger sett densities (see Judge *et al.* ^9^ for further details) This was reflected in the present study, with sample squares concentrated in LCGs with historically higher badger social group densities.

We randomly selected 1 km squares from those containing at least one main sett in the most recent badger sett survey of England and Wales^9^. The number of sampled squares in each LCG was determined by estimating the relative standard deviation of the mean number of badgers within and between social groups, based on the weighted mean and weighted pooled standard deviation of social group size reported in the available literature^23–27^. Assuming that each square only contained one main sett, the target distribution of hair-trapped squares across the LCGs, which had a Relative Standard Error (RSE) of 8.5%, is shown in Table 4. Due to issues with obtaining landowner permission and time constraints the actual number of squares that were sampled with hair traps was 72, however as 15 squares contained two main setts and three had three main setts, hair traps were ultimately placed at a total of 120 main setts (Table 4), reducing the RSE to 8.2%.

**Table 4:** Land Class Groups showing the associated total number of squares with main setts recorded in the 2011–13 badger sett survey of England and Wales^9^ and the numbers of squares and main setts that were hair-trapped to estimate social group size in the present study.

Land Class Group	Number of squares with main setts	Target number of squares to hair trap	Actual number of squares hair trapped	Number of main setts hair trapped
1	110	17	13	14
2	132	17	12	16
3	7	2	3	3
4	261	38	27	66
5	60	10	8	9
6	42	6	9	12
7	0	0	0	0

As no main setts were recorded in Land Class Group 7 in the 2011–13 badger sett survey, it was not possible to deploy any hair traps in this Group.

During the 2011–13 badger sett survey of England and Wales^9^ no main setts were recorded in any of the 29 squares surveyed in LCG7, although other types of sett were observed. Therefore, we assumed that the number of badgers per social group in LCG7 was the same as in LCG6 as they are both upland landscapes. Furthermore, as LCG7 represents only 2.9% of the rural land area in England and Wales and has a very low density of badger setts, it is considered very unlikely that any significant additional error would be introduced into the national estimates under these assumptions.

### Hair Trapping

Hair traps were deployed at all main setts within the selected sample of 1 km squares. On the initial visit an activity score (number of well used, partially used and disused holes) was recorded for each sett. Fieldwork took place from October 2012 to February 2013 and October 2013 to March 2014 inclusive. This overlapped and closely followed the sett survey fieldwork which took place between November 2011 and March 2013^9^. Hair traps were only deployed at main setts to minimise the possibility of sampling badgers from neighbouring social groups. Fieldwork was not carried out in the summer as there is some evidence to suggest that this is when badgers spend more time away from the main sett^28, 29^. Also, hair trapping data collected in the summer would include potentially significant numbers of newly emerged cubs, which have a relatively high mortality rate^30^, and may increase the variance of group size estimates.

**Figure 2:**
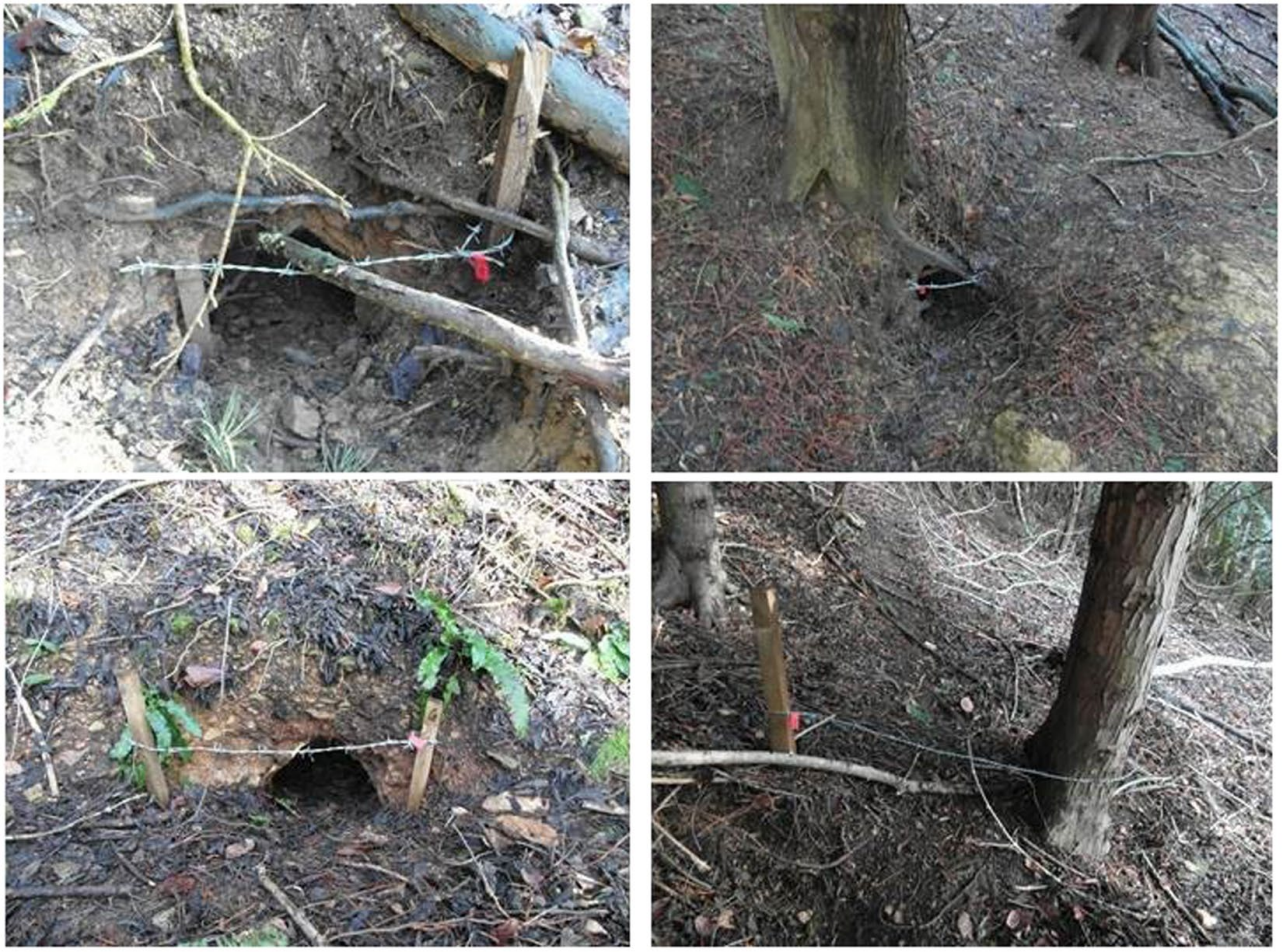
Examples of hair traps *in situ* over badger sett entrance holes and runs.

Hair traps were deployed and hairs collected following a methodology developed for previous applications of this approach for estimating badger numbers^31, 32^. Hair traps consisted of a strand of barbed wire suspended across sett entrances and/or ‘runs’ close to the sett using natural features where possible or wooden stakes if necessary (Fig. 2). The number of traps per sett varied dependent on the number of active entrances and runs present, and was determined by an experienced surveyor.

Each hair trap was labelled with a unique identifier. Hair traps remained *in situ* for at least four weeks and during this time were visited for hair collection on alternate days, resulting in 14 sample collection days for each sett. On each visit all the hairs on each trap were collected into a labelled bag. The sample of hairs in each bag therefore represented a specific hair trap-day combination.

### Genetic typing

Genotyping was carried out by the Food and Environment Research Agency, York, U.K. following the protocols used in previous studies^31, 32^. A single hair was taken from each bag (i.e. from each hair trap-day) for genotyping. This hair was selected by visual assessment of the quality of the hair follicle, and with preference given to longer hairs, as experience suggests that these characteristics maximise the probability of obtaining a genetic profile. DNA extraction from hair samples was carried out using a Chelex c protocol^31^. DNA samples were amplified for ten microsatellite loci. Null alleles (alleles which failed to amplify reliably for a particular microsatellite, leading to errors in the final data) were estimated using the programme CERVUS 3.0.333^33^.One marker was excluded from subsequent analyses as it was associated with a high estimated proportion of null alleles. Only complete microsatellite profiles (i.e. only samples which had correctly amplified for all remaining nine markers) were used in subsequent analyses. Using a panel of ten microsatellites has a probability of producing a false match between two randomly selected individuals of less than 1 in 1,000 million. As the individuals within a badger social group are highly likely to be related, they have a greater chance of sharing the same profile. In order to quantify the likelihood of observing a false match within the sample using nine microsatellites, the combined non-exclusion probabilities for all individuals (P_*ident*_) and siblings (P_*sib*_) were calculated for the nine microsatellites as P_*ident*_ = 7.60E-9 and P_*sib*_ = 0.00055103. Therefore, there was a less than 0.1% chance of observing a single false match between profiles produced by 98 pairs of siblings.

Each profiled hair was allocated to an individual badger using the programme GeneCap^34^. All identical profiles were assigned to a single individual. In general, the likelihood of two individuals sharing the same profile with the exception of a single mutation was lower than the likelihood of a single individual appearing to have two profiles due to a sequencing/scoring error (‘stutter’ or allelic drop-out). Hence, profiles which differed from one another by only a single mutation were assigned to a single individual. However, profiles with single-mutation differences that were not easily explained by sequencing error were assigned to separate individuals.

### Reassignment of individuals to social groups

Eight badgers were identified from hair traps at more than one sett in the same square. A desk-based assessment was undertaken by experts using frequency caught at each sett, expert opinion, reference to the activity scores (recorded when the setts were visited to install hair traps) and original survey results. This resulted in six individuals being classified as resident at one main sett and ‘visiting’ the other. In the remaining two cases, the setts were determined not to be main setts because of the level of activity observed at the time of hair trapping and their distance to, and level of activity at, the nearest active sett. These reclassifications were attributed to changes in sett use between the original survey and when hair traps were deployed.

### Statistical Analyses

Estimation of the number of individuals in each social group was undertaken using “Capwire” a method designed for estimating the size of small populations from genetic mark-recapture data^35^. The number of badgers associated with each social group (i.e. with each main sett) was estimated by a mark-recapture analysis of the ‘capture’ of hairs from individual badgers. The number of badgers at each main sett was estimated using the two intrinsic rates model^35^.

The method for estimating the number of individuals in social groups was modified when there were insufficient observations to allow estimation of the model parameters (n = 29 setts), as follows;

i. A sett was excluded from the estimate if all the badgers at a sett were only hair trapped once because it was not possible to confidently assign those animals as being resident at that sett (n = 13 setts).
ii. If genotyping indicated that all badgers were hair trapped the same number of times at a given sett (as long as the number of captures for each animal was greater than one), the group size estimate was deemed to be the same as the number of individuals identified by hair trapping (n = 4 setts).
iii. If there were only two capture rates at a sett (e.g. some individuals were hair trapped once and the rest twice, or some twice and the rest four times etc.) then group size was estimated under the assumption that the underlying capture rate across individuals in the group was constant (n = 12 setts).

Scenarios ii. and iii. may be expected to produce estimates which are, on average smaller than the true social group size. Therefore our estimates for 16 of the 89 (18%) social groups may have been underestimated. Sizes were estimated independently for each social group, and hence capture rates and the effectiveness of hair trapping were assumed to be specific to each group.

### Estimation of the mean social group size in each Land Class Group

Two sources of uncertainty about the mean group size were considered whilst estimating the mean social group size in each LCG: (i) the uncertainty associated with the estimate of the size of each social group, and (ii) the variation in size between different social groups within an LCG. To quantify these sources of uncertainty:

1. Each social group size was represented by a single parametric bootstrap sample.
2. Each LCG was represented by a simple bootstrap sample (re-sampling with replacement) of social groups within that LCG.
3. A mean number of badgers per social group was calculated for the sample provided by each LCG.
4. Steps 1–3 were repeated 1000 times. The standard uncertainty of the estimated mean social group size for each LCG was provided by the standard deviation of the 1000 estimates. This provided a measure of the effect of both between social-group variation and uncertainty associated with social group sizes within that LCG.

Although we may expect the variation in the number of individuals per social group to be best described by a distribution that is consistent with over-dispersed counts, such as a negative binomial, the uncertainty associated with mean social group size in each LCG was found to be sufficiently well approximated by a normal distribution based on the observed distribution of bootstrap estimates. Hence, confidence intervals for the mean number of badgers per social group were based on quantiles of normal distributions, and standard errors for population estimates based on combining Land Class Groups were based on a first-order Taylor series. Land Class Group 3 was an exception, but because of the very low number of social groups estimated to be present in this Group this has a negligible impact on the estimate.

### Model Fit

Model fit for each social group was assessed by comparing the parametric bootstrap estimates for that social group with the central estimate of group size. An estimate outside the range of parametric bootstrap estimates was considered to be indicative of a case where the model used to estimate the size of the social group did not fit well with the observations. All estimates were within the expected interval based on bootstrapping. However, in social groups where a large proportion of observations were of single capture individuals, the confidence intervals were wide, demonstrating that the individual estimates of social group size are very sensitive to low capture rates.

### Estimation of the number of badgers in England and Wales

The number of badgers in each LCG was calculated as the product of the mean number of badgers per social group estimated in this study, and the number of social groups estimated in the 2011–13 sett survey^9^. The error associated with the estimate was based on an assumption that estimates of social group size and numbers of social groups were unbiased and the errors associated with estimates were uncorrelated.

## Electronic supplementary material

Supplementary Info

## References

[1] Donnelly CA (2007). Impacts of widespread badger culling on cattle tuberculosis: concluding analyses from a large-scale field trial. International Journal of Infectious Diseases.

[2] Moore N (1999). Survey of badger *Meles meles* damage to agriculture in England and Wales. Journal of Applied Ecology.

[3] Delahay RJ (2009). Managing conflict between humans and wildlife: trends in licensed operations to resolve problems with badgers *Meles meles* in England. Mammal Review.

[4] Roper, T. *Badger*. Harper Collins UK (2010).

[5] Cresswell, P., Harris, S. & Jefferies, D. J. *The history, distribution, status and habitat requirements of the badger in Britain*. Nature Conservancy Council, Peterborough, UK (1990).

[6] Wilson, G., Harris, S. & McLaren, G. *Changes in the British badger population, 1988 to 1997*. People’s Trust for Endangered Species (1997).

[7] Rainey, E. *et al.**Scottish Badger Distribution Survey 2006–2009-estimating the density and distribution of badger main setts in Scotland* (2009).

[8] Reid N, Etherington TR, Wilson GJ, Montgomery WI, McDonald RA (2012). Monitoring and population estimation of the European badger *Meles meles* in Northern Ireland. Wildlife Biology.

[9] Judge, J., Wilson, G. J., Macarthur, R., Delahay, R. J. & McDonald, R. A. Density and abundance of badger social groups in England and Wales in 2011–2013. *Sci. Rep.* **4**, doi:10.1038/srep03809 (2014). 10.1038/srep03809 PMC3899851 24457532

[10] Clements ED, Neal EG, Yalden DW (1988). The national badger sett survey. Mammal Review.

[11] Neal, E. & Cheeseman, C. *Badgers*. T & AD Poyser (1996).

[12] Krebs, J. R. *et al.**Bovine tuberculosis in cattle and badgers*. MAFF Publications, London, UK (1997).

[13] Rogers LM, Cheeseman CL, Mallinson PJ, Clifton-Hadley R (1997). The demography of a high-density badger (*Meles meles*) population in the west of England. Journal of Zoology.

[14] Delahay R (2013). Long-term temporal trends and estimated transmission rates for *Mycobacterium bovis* infection in an undisturbed high-density badger (*Meles meles*) population. Epidemiology and infection.

[15] Ostler JR, Roper TJ (1998). Changes in size, status, and distribution of badger *Meles meles* L. setts during a 20-year period. Zeitschrift fur Säugetierkunde.

[16] Macdonald DW, Newman C (2002). Population dynamics of badgers (*Meles meles*) in Oxfordshire, U.K.: numbers, density and cohort life histories, and a possible role of climate change in population growth. Journal of Zoology.

[17] Kruuk H, Parish T (1987). Changes in the size of groups and ranges of the European badger (*Meles meles*) in an area in Scotland. Journal of Animal Ecology.

[18] Bunce, R. G. H., Barr, C. J. & Whittaker, H. A. Land classes in Great Britain: preliminary descriptions for users of the Merlewood method of land classification. *Merlewood Research and Development Paper no 86*, Institute of Terrestrial Ecology (1981).

[19] Bunce RGH, Barr CJ, Gillespie MK, Howard DC (1996). The ITE Land classification: Providing an environmental stratification of Great Britain. Environmental Monitoring and Assessment.

[20] Walsh AL, Harris S (1996). Factors determining the abundance of vespertilionid bats in Britain: geographical, land class and local habitat relationships. Journal of Applied Ecology.

[21] Walsh AL, Harris S (1996). Foraging habitat preferences of vespertilionid bats in Britain. Journal of Applied Ecology.

[22] Hutchings, M. R. & Harris, S. The current status of the brown hare (*Lepus europaeus*) in Britain. *Report to JNCC* University of Bristol, Bristol (1996).

[23] Delahay RJ, Carter SP, Forrester GJ, Mitchell A, Cheeseman CL (2006). Habitat correlates of group size, bodyweight and reproductive performance in a high-density Eurasian badger (*Meles meles*) population. Journal of Zoology.

[24] Hewitt SE, Macdonald DW, Dugdale HL (2009). Context-dependent linear dominance hierarchies in social groups of European badgers, *Meles meles*. Animal Behaviour.

[25] Johnson DDP, Kays R, Blackwell PG, Macdonald DW (2002). Does the resource dispersion hypothesis explain group living?. Trends in Ecology & Evolution.

[26] Palphramand KL, Newton-Cross G, White PC (2007). Spatial organization and behaviour of badgers (*Meles meles*) in amoderate-density population. Behavioral Ecology and Sociobiology.

[27] Woodroffe R (2009). Social group size affects *Mycobacterium bovis* infection in European badgers (*Meles meles*). Journal of Animal Ecology.

[28] Roper TJ, Ostler JR, Schmid TK, Christian SF (2001). Sett use in European badgers *Meles meles*. Behaviour.

[29] Weber N (2013). Denning behaviour of the European badger (*Meles meles*) correlates with bovine tuberculosis infection status. Behavioral Ecology and Sociobiology.

[30] Cheeseman CL, Wilesmith JW, Ryan J, Mallinson PJ (1987). Badger population dynamics in a high-density area. Symposia of the Zoological Society of London.

[31] Frantz AC (2004). Estimating population size by genotyping remotely plucked hair: the European badger. Journal of Applied Ecology.

[32] Scheppers TL (2007). Estimating social group size of Eurasian badgers *Meles meles* by genotyping remotely plucked single hairs. Wildlife Biology.

[33] Kalinowski ST, Taper ML, Marshall TC (2007). Revising how the computer program Cervus accommodates genotyping error increases success in paternity assignment. Molecular Ecology.

[34] Wilberg MJ, Dreher BP (2004). Genecap: a program for analysis of multilocus genotype data for non-invasive sampling and capture-recapture population estimation. Molecular Ecology Notes.

[35] Miller CR, Joyce P, Waits LP (2005). A new method for estimating the size of small populations from genetic mark-recapture data. Molecular Ecology.

