# Supplementary material for: Abundance of badgers (Meles meles) in England and Wales
Source: Sci Rep. 2017 Mar 21;7:276. doi: 10.1038/s41598-017-00378-3 (PMC5428277; doi:10.1038/s41598-017-00378-3)
Supplement: Supplementary file 1 — Supplementary Info [file 41598_2017_378_MOESM1_ESM.pdf]

Abundance of badgers (*Meles meles*) in England and Wales

Johanna Judge, Gavin J. Wilson, Roy Macarthur, Robbie A McDonald and Richard J Delahay

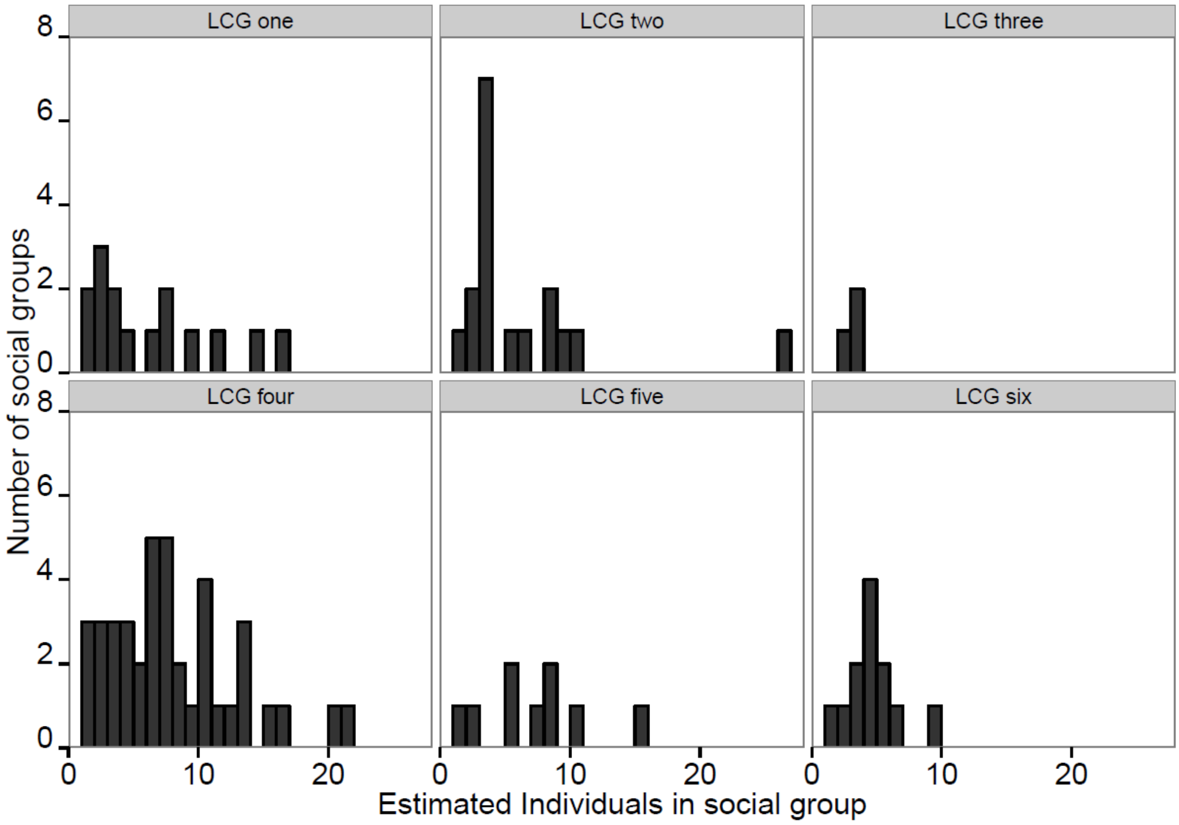

Figure S1. Frequency distributions showing the estimated numbers of badgers per social group in each of the Land Class Groups 1 – 6. LCG7 was not included in this survey because no main setts were recorded in the 2011 – 2013 sett survey.
